# Guiding properties of asymmetric hybrid plasmonic waveguides on dielectric substrates

**DOI:** 10.1186/1556-276X-9-13

**Published:** 2014-01-10

**Authors:** Wei Wei, Xia Zhang, Yongqing Huang, Xiaomin Ren

**Affiliations:** 1 State Key Laboratory of Information Photonics and Optical Communications, Beijing University of Posts Telecommunications, P. O. Box 66, Beijing 100876, China

**Keywords:** Surface plasmons, Hybrid plasmonic waveguides, Integrated photonic devices

## Abstract

We proposed an asymmetric hybrid plasmonic waveguide which is placed on a substrate for practical applications by introducing an asymmetry into a symmetric hybrid plasmonic waveguide. The guiding properties of the asymmetric hybrid plasmonic waveguide are investigated using finite element method. The results show that, with proper waveguide sizes, the proposed waveguide can eliminate the influence of the substrate on its guiding properties and restore its broken symmetric mode. We obtained the maximum propagation length of 2.49 × 10^3^ μm. It is approximately equal to that of the symmetric hybrid plasmonic waveguide embedded in air cladding with comparable nanoscale confinement.

## Background

Surface plasmons (SP) are optically induced oscillations of free electrons at the surface of a metal and can localize the guided light far beyond the diffraction limit for electromagnetic waves in dielectric media [1,2]. This could lead to miniaturized photonic circuits with a length scale much smaller than those currently achieved [3,4]. Various kinds of plasmonic waveguides including metal grooves [5,6], a chain of metal particles [7], metal stripes [8], and metal nanowires [9-11] have been proposed and investigated to realize highly integrated photonic circuits [7-12]. However, due to ohmic loss of metal [13], the propagation lengths of guided modes in plasmonic waveguides are typically short under tight confinement, which greatly limits the scope for practical applications. The main limitation of such waveguides is the trade-off between confinement and loss. Two promising approaches, the symmetric SP mode and hybrid SP mode, are proposed to optimize the balance between propagation length and mode confinement: (1) the symmetric SP mode exhibits a lower attenuation than its asymmetric counterpart, and therefore, it is sometimes referred as to long-range SP [8]; (2) in a hybrid SP mode plasmonic waveguide, the coupling between plasmonic and waveguide modes across the gap enables ‘capacitor-like’ energy storage that allows subwavelength light propagation in nonmetallic regions with strong mode confinement [14]. Therefore, symmetric hybrid plasmonic (SHP) waveguides combining the two ideas of symmetric and hybrid SP modes can exhibit a quite long propagation length with strong mode confinement [15-20].

For practical implementations, an SHP waveguide needs to be placed on a substrate. The presence of the substrate breaks the symmetry of SP mode, leading to the dramatic decrease of propagation length. Here in this paper, by introducing an asymmetry into the SHP waveguide, we propose a novel asymmetric hybrid plasmonic (AHP) waveguide to eliminate the influence of a substrate on its guiding properties and restore its broken symmetric SP mode. Based on the combination of symmetric and hybrid SP modes, the AHP waveguide exhibits a quite long propagation length along with nanoscale mode confinement. In the following sections, with the finite element method (FEM), we investigate the guiding properties of the AHP waveguide on a substrate at a wavelength of 1,550 nm to target potential applications in telecommunications. Compared to an SHP waveguide with the same structure embedded in air cladding, the propagation length of the AHP waveguide is approximately the same along with a comparable normalized modal area. Moreover, the AHP waveguide has a horizontal slot structure featured with a horizontal low index slot, which can be convenient to be fabricated by layered deposition or thermal oxidation [21].

## Methods

**Figure 1:**
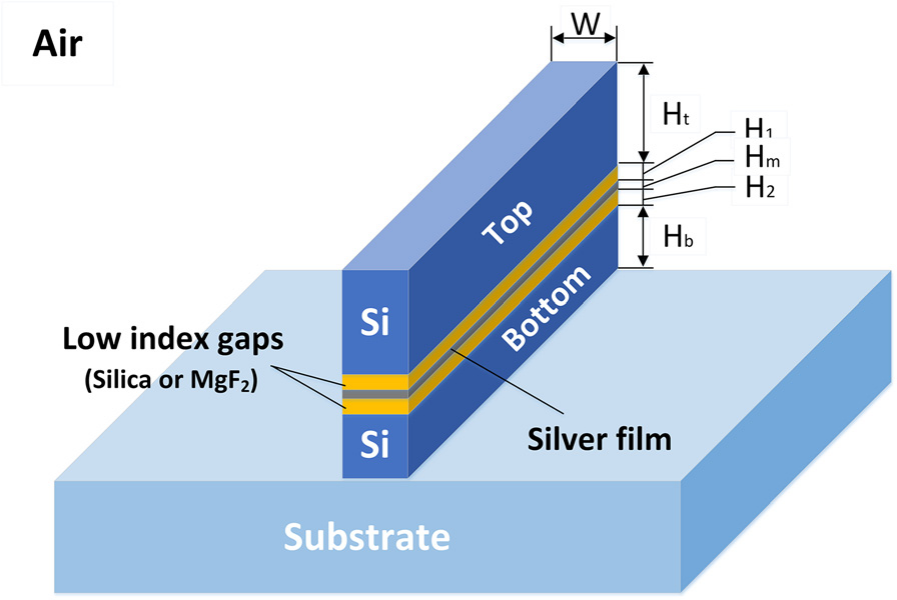
Schematic of the proposed AHP waveguide.

The schematic of the AHP waveguide on a silica substrate is demonstrated in Figure 1, where two layers of dielectrics (SiO_2_-Si) are placed on both sides of a thin silver film. The silver film has a height of *H*_m_. The heights of the low index gaps are denoted by *H*_1_ and *H*_2_, respectively. Silica is chosen to form low index gaps with respect to silicon. The heights of the top and bottom silicon layers are denoted by *H*_t_ and *H*_b_, respectively. All these metallic and dielectric sections on the silica substrate have the same width of *W*. In an SHP waveguide, *H*_t_ and *H*_1_ are equal to *H*_b_ and *H*_2_, respectively. However, in the AHP waveguide, *H*_b_ is smaller than *H*_t_, resulting in an asymmetry in the SHP waveguide. The optical properties of the AHP waveguide are investigated using FEM at 1,550 nm. The refractive index of silver is taken from [22]. To calculate the normalized modal area and propagation length of the AHP waveguide, we introduce Equations 1, 2, and 3 [14]:

(1) $$A_{m}=\frac{W_{m}}{\max\left\{W\left(r\right)\right\}}=\frac{1}{\max\left\{W\left(r\right)\right\}}\iint_{\infty }W\left(r\right)d^{2}r$$

where *W*_*m*_ is the total mode energy and *W*(*r*) is the energy density (per unit length flowed along the direction of propagation). For dispersive and lossy materials, the *W*(*r*) inside can be calculated as Equation 2:

(2) $$W\left(r\right)=\frac{1}{2}\left(\frac{d\left(\epsilon \left(r\right)\omega \right)}{\mathrm{d\omega}}{\left|E\left(r\right)\right|}^{2}+\mu_{0}{\left|H\left(r\right)\right|}^{2}\right).$$

The normalized modal area is defined as *A*_*m*_*/A*_0_ to quantitatively evaluate the mode confinement, where *A*_0_ represents the diffraction-limited area in free space, *A*_0_ *= λ*^2^/4. The propagation length is defined as Equation 3:

(3) L=12Imβ.

## Results and discussion

In the first section, we investigate the guiding properties and optimize structure parameters of the SHP waveguide on a silica substrate via calculating the propagation length and normalized modal area. For further practical applications, the structure parameters of the SHP waveguide in the ideal condition (embedded in air cladding) are not investigated in detail here. We only compare the guiding properties between the AHP waveguide on a substrate and the SHP waveguide embedded in air cladding with the same structure parameters as the AHP waveguide. Then, in the second section, we propose the AHP waveguide by introducing an asymmetry into the SHP waveguide. Electromagnetic energy density profiles of an SHP waveguide embedded in air cladding, on a silica substrate, and an AHP waveguide on a silica substrate are demonstrated to compare SP mode distributions. We also investigate the guiding properties of the AHP waveguide as the height of mismatch varies. Here, it is worth mentioning that some values of the geometry parameters of the AHP waveguide considered in the study are reaching the limit where the local solutions of macroscopic Maxwell's equations may be not accurate enough for the descriptions of the electromagnetic properties. For more rigorous investigations, one needs to take nonlocal effects into account [14,23,24].

### SHP waveguide on a substrate

**Figure 2:**
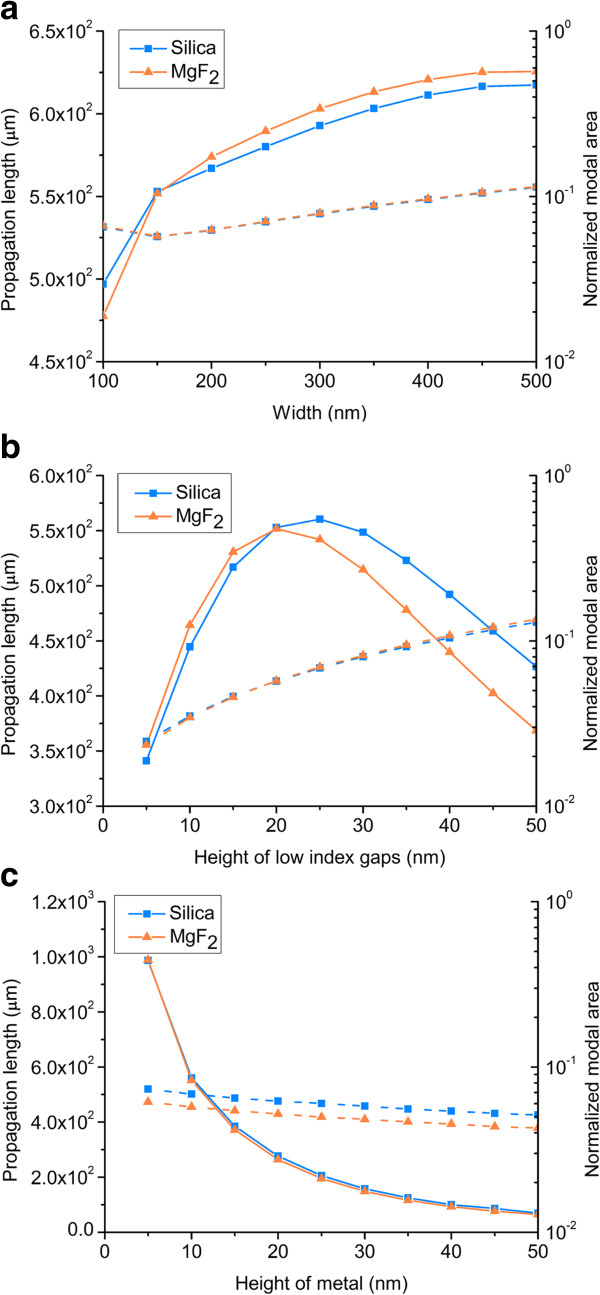
**Propagation length and normalized modal area.** They are shown versus **(a)** width of the waveguide, **(b)** height of low index gaps, and **(c)** height of metal stripe.

Propagation length and normalized modal area are important parameters describing the mode features in a plasmonic waveguide. For applicable conditions, the SHP waveguide is always on a substrate rather than being embedded in air cladding. Therefore, in this section, we investigate the geometric dependence of the propagation length and normalized modal area of the SHP waveguide on a substrate. The propagation lengths and normalized modal areas versus the width of the low index gaps are shown in Figure 2a, where *H*_1_, *H*_2_, *H*_m_ and *H*_t_, *H*_b_ are fixed at 20, 10, and 300 nm, respectively. The propagation lengths of silica and MgF_2_ increase as the width becomes wider. When the width increases, the refractive index difference brought by the substrate, which breaks the symmetric modal distribution, becomes smaller. Therefore, the propagation length increases. However, the size of waveguide increases dramatically while the propagation length increases relatively tenderly. When the width is 150 nm, there are minimum values in curves of the normalized modal area for both silica and MgF_2_. At this point, the electromagnetic energy of SP mode is mostly confined in the waveguide. Due to the fact that the smallest normalized modal areas are obtained at a width of 150 nm, in the following calculations, we fix the width at 150 nm. The propagation lengths and normalized modal areas versus the height of low index gaps for silica and MgF_2_ are shown in Figure 2b. It is obvious that the normalized modal areas increase almost linearly with the increased heights of the low index gaps. The curves of propagation lengths are both parabolic. The propagation lengths reach the maximum values when the heights of low index gaps are equal to 25 and 20 nm, respectively. The electromagnetic energy of SP mode is mainly confined and guided in the low index gaps of the SHP waveguide. With the height of the low index gaps increasing in the rising area of the curves, more proportions of mode are confined in the gaps, which results in an extended propagation length. In this case, the mode is a hybrid mode that features both dielectric and SP characteristics [14]. With the height of the low index gaps increasing in the dropping area of the curves, the confinement becomes weaker and less proportions of mode are confined in the low index gaps, resulting in an increased loss. In the following calculations, to obtain the optimal performance of the SHP waveguide, we fix the height of low index gaps for silica and MgF_2_ at 25 and 20 nm, respectively. In Figure 2c, we demonstrate the propagation lengths and normalized modal areas versus the height of metal for silica and MgF_2_ of the low index gaps. The propagation lengths and normalized modal areas both decrease as the height of metal increases. This can be explained as that when the height of metal becomes wider, more proportions of mode are confined in the metal, leading to increased loss and normalized modal area. Therefore, in the following, we fix the height of metal at 5 nm, emphatically considering the propagation length. Considering an ideal condition of the silica SHP waveguide being embedded in air cladding with structure parameters the same as that mentioned before, the calculated propagation length and normalized modal area are 2.38 × 10^3^ μm and 0.076, respectively. Thus, compared to the guiding properties of the SHP embedded in air cladding, the presence of the substrate has a bad influence on the guiding properties of the SHP on the substrate. Next, we will eliminate the influence of the substrate on the guiding properties of the SHP on the substrate in an effective way.

### AHP waveguide on a substrate

**Figure 3:**
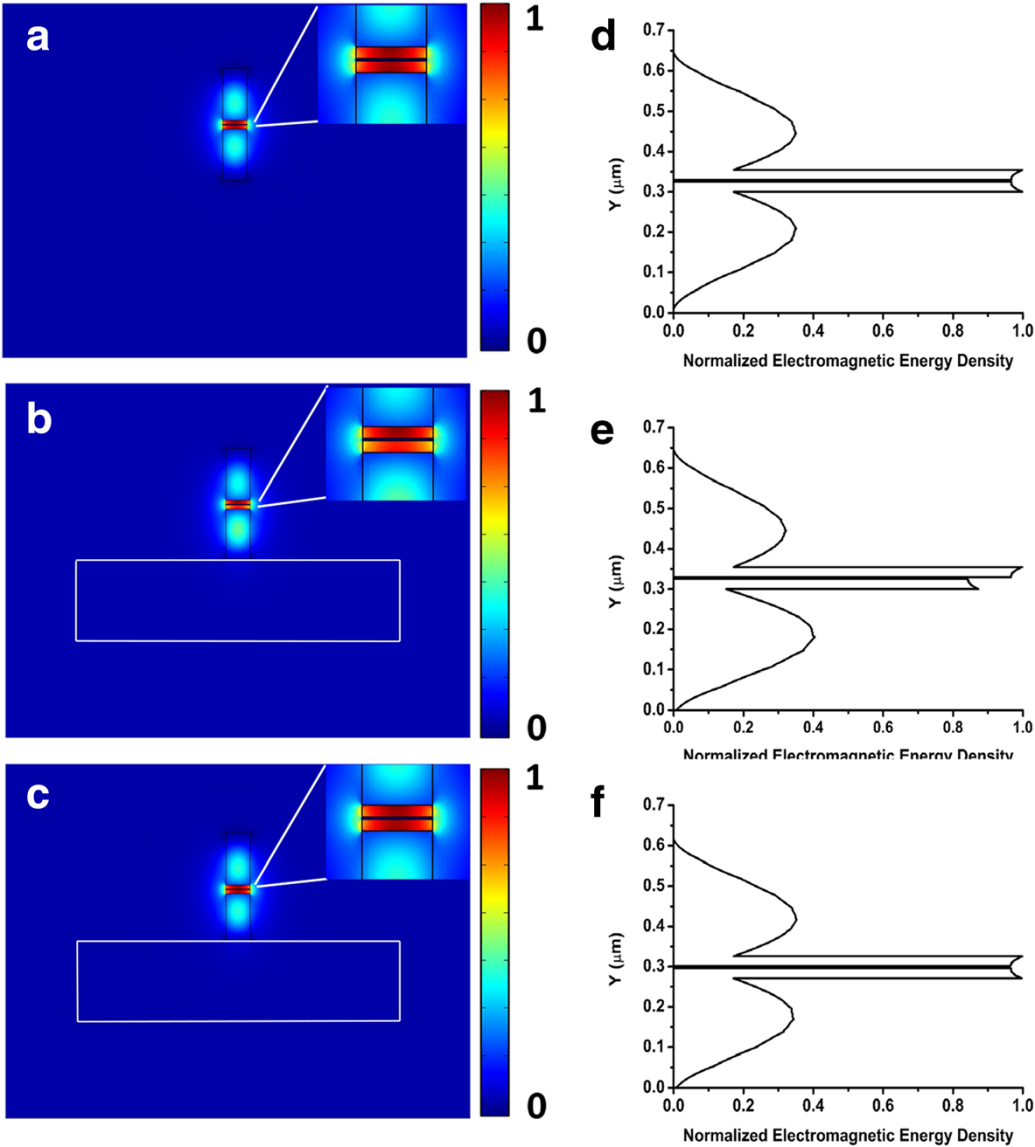
**Electromagnetic energy density profiles of the SHP and AHP waveguides.** The profiles are SHP waveguides **(a)** in air and **(b)** on a silica substrate, and **(c)** AHP waveguides on silica substrate. **(d, e, f)** Corresponding normalized electromagnetic energy densities along the *Y*-axis (from 0 to 0.6 μm) are shown.

In this section, the structure parameters of the waveguide are the same as those in the previous section. Electromagnetic energy density profiles of the SHP waveguide in air, on a silica substrate, and an AHP waveguide on a silica substrate are shown in Figure 3a,b,c, respectively. In Figure 3a, the electromagnetic energy density profile of the SHP waveguides embedded in air cladding is symmetric. The SP mode is strongly confined and guided in two dimensions within the low index gaps, which is bounded by the high index material and metal. However in Figure 3b, the presence of a silica substrate breaks the symmetry of the electromagnetic energy density of the SHP waveguide. The electromagnetic energy density distributes towards the upper low index gap of the SHP waveguide. When we introduce an asymmetry into the SHP waveguide on a silica substrate by decreasing *H*_b_, the asymmetric mode becomes symmetric as shown in Figure 3c. The AHP waveguide has an asymmetric structure, but its electromagnetic energy density distribution is symmetric. The asymmetric structure of the AHP waveguide restores the symmetry of the SP mode.

**Figure 4:**
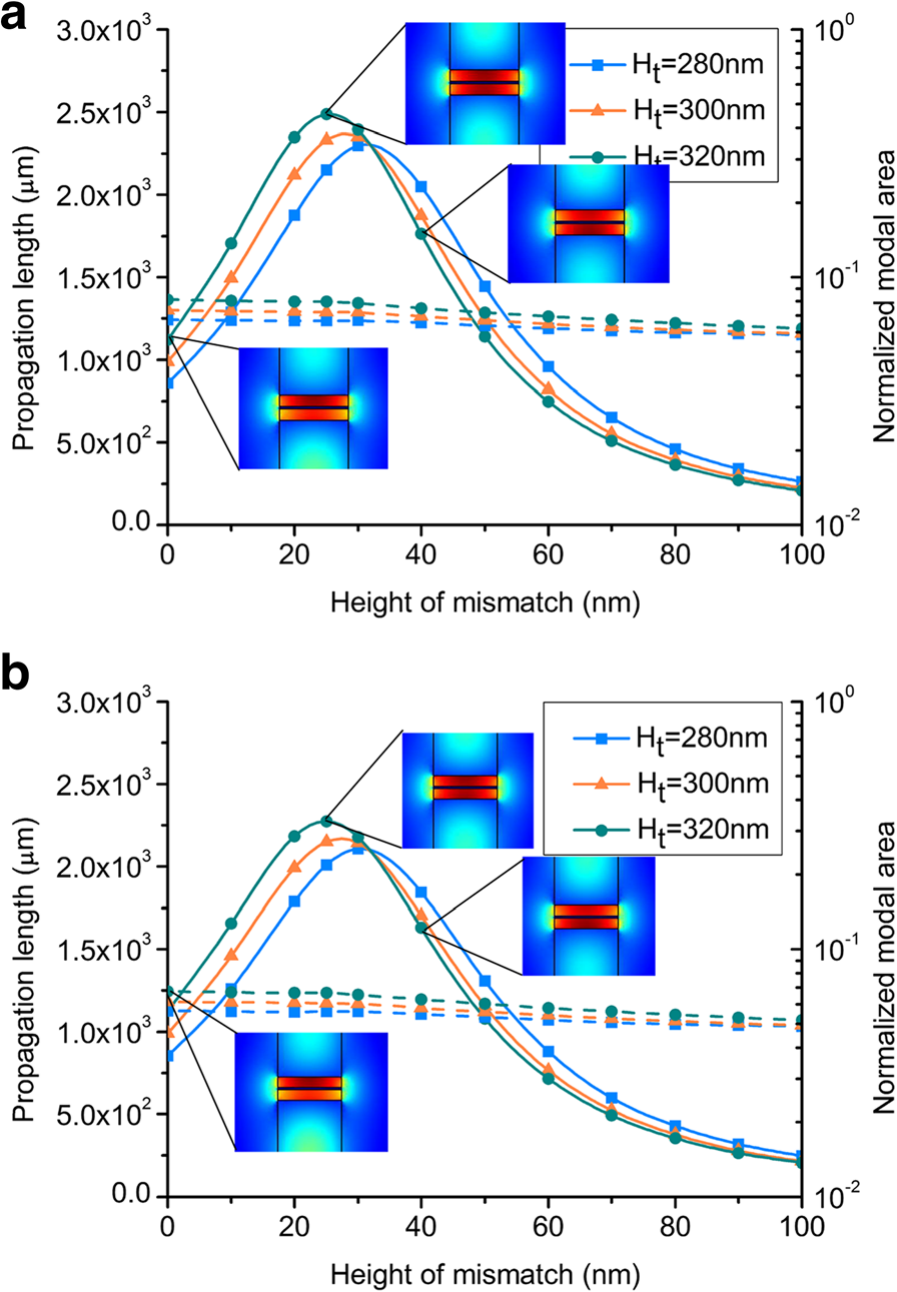
**Propagation length and normalized modal area of silica- and MgF**_**2**_**-based AHP waveguide versus height of mismatch. (a)** Silica- and **(b)** MgF_2_-based AHP waveguide. The insets indicate electromagnetic energy density profiles of different heights of mismatch.

The height of mismatch is defined as Δ = *H*_t_ - *H*_b_ to describe the asymmetry of the AHP waveguide. The propagation length and normalized modal area of both silica and MgF_2_ AHP waveguides versus the height of mismatch are shown in Figure 4, under the conditions of three different values of *H*_t_. As shown in Figure 4a, when the height of mismatch varies from 0 to 100 nm, the normalized modal area changes a little in the range of 0.06 to 0.08, which is far below the diffraction limit [25]. In a hybrid plasmonic waveguide, most proportions of the SP mode are confined in the low index gap [14]. Thus, introducing an asymmetry to the structure by varying the height of mismatch has little effect on the normalized modal area. The curves of propagation length are nearly parabolic, and the propagation length increases with the increase of *H*_t_. As the insets of *H*_t_ = 320 nm as shown in Figure 4a, the electromagnetic energy of SP mode is asymmetric at Δ = 0 nm. With the increase of the height of mismatch, the asymmetric mode becomes symmetric at Δ = 25 nm. At this time, the propagation length reaches its maximum value. Then, with the increase of the height of mismatch, the symmetric mode becomes asymmetric again at Δ = 40 nm, leading to the decrease of the propagation length. As the normalized modal areas is ultrasmall for different *H*_t_ values, we obtain the maximum propagation length of 2.49 × 10^3^ μm for *H*_t_ = 320 nm. The propagation length of the AHP waveguide increases 122% than that of the SHP waveguide on a substrate. Compared to the ideal condition of the SHP in air cladding, the propagation length of the AHP waveguide is approximately equal to that of the SHP waveguide in air (2.38 × 10^3^ μm) with a comparable normalized modal area. Thus, the introduced asymmetry to the structure of the SHP waveguide is vital to the extension of the propagation length while exerting little effect on the normalized modal area. The phenomenon in Figure 4b is similar to that in Figure 4a, but the performance of the silica-based AHP waveguide is better than that of the MgF_2_-based AHP waveguide.

## Conclusions

In conclusion, we reveal that the AHP waveguide combining the advantages of symmetric (long-range) SP mode and hybrid plasmonic waveguides is capable of supporting long-range propagation of the guided waves with nanoscale mode confinement. The proposed structure is realized by introducing an asymmetry into the SHP waveguide. Theoretical calculations show that the AHP waveguide can eliminate the effect of a silica substrate on the guiding properties of the SHP waveguide and restores the symmetry of SP mode. Thus, the AHP waveguide on a substrate can perform the same as the SHP waveguide embedded in air cladding. Considering different materials of the low index gaps in the AHP waveguide, the performance of the silica-based AHP waveguide is better than the MgF_2_-based AHP waveguide. The proposed AHP waveguide can be conveniently fabricated by existing technologies like layered deposition or thermal oxidation. This may have practical applications in highly integrated circuits as plasmonic interconnects.

## Abbreviations

AHP: Asymmetric hybrid plasmonic; FEM: Finite element method; SHP: Symmetric hybrid plasmonic; SP: Surface plasmons.

## Competing interests

The authors declare that they have no competing interests.

## Authors’ contributions

WW proposed the asymmetric idea, calculated properties of the proposed waveguide, and wrote the manuscript. XZ, YH, and XR analyzed the data and revised the manuscript. All authors read and approved the final manuscript.

## References

[B1] Polman A Applied physics plasmonics applied Science 2008 9 868 869 10.1126/science.1163959 18988831

[B2] Gramotnev DK Bozhevlnyi SI Plasmonic beyond the diffraction limit Nature Photon 2010 9 83 91 10.1038/nphoton.2009.282

[B3] William LB Alain D Thomas WE Surface plasmon subwavelength optics Nature 2003 9 824 830 10.1038/nature01937 12917696

[B4] Ozbay E Plasmonics: merging photonics and electronics at nanoscale dimensions Science 2006 9 189 193 10.1126/science.1114849 16410515

[B5] Bozhevolnyi SI Volkov VS Devaux E Laluet JY Ebbesen TW Channel plasmon subwavelength waveguide components including interferometers and ring resonators Nature 2006 9 508 511 10.1038/nature04594 16554814

[B6] Bian YS Zheng Z Zhao X Su YL Liu L Liu JS Zhu JS Zhou T Highly confined hybrid plasmonic modes guided by nanowire-embedded-metal grooves for low-loss propagation at 1,550 nm IEEE J Sel Topics Quantum Electron 2013 9 4800106

[B7] Quinten M Leitner A Krenn JR Aussenegg FR Electromagnetic energy transport via linear chains of silver nanoparticles Opt Lett 1998 9 1331 1333 10.1364/OL.23.001331 18091775

[B8] Burke JJ Stegeman GI Surface-polariton-like waves guided by thin, lossy metal films Phys Rev B 1986 9 5186 5201 10.1103/PhysRevB.33.5186 9939016

[B9] Manjacavas A de Aabajc FJ G Robust plasmon waveguides in strongly interacting nanowire arrays Nano Lett 2009 9 1285 1289 10.1021/nl802044t 18672946

[B10] Zhang ZX Hu ML Chan KT Wang CY Plasmonic waveguiding in a hexagonally ordered metal wire array Opt Lett 2010 9 3901 3903 10.1364/OL.35.003901 21124559

[B11] Wei W Zhang X Yu H Huang YQ Ren XM Plasmonic waveguiding properties of the gap plasmon mode with a dielectric substrate Photon Nano Fund Appl 2013 9 279 287 10.1016/j.photonics.2013.06.006

[B12] Chen L Li X Wang GP Li W Chen SH Xiao L Gao DS A silicon-based 3-D hybrid long-range plasmonic waveguide for nanophotonic integration J Lightw Tech 2012 9 163 168

[B13] Zayats AV Smolyaninov II Maradudin AA Nano-optics of surface plasmon polaritons Phys Rep 2005 9 131 314 10.1016/j.physrep.2004.11.001

[B14] Oulton RF Sorger VJ Genov DA Pile DFP Zhang X A hybrid plasmonic waveguide for subwavelength confinement and long-range propagation Nat Photon 2008 9 496 500 10.1038/nphoton.2008.131

[B15] Bian YS Zheng Z Zhao X Zhu JS Zhou T Symmetric hybrid surface plasmon polariton waveguides for 3D photonic integration Opt Express 2009 9 21320 21325 10.1364/OE.17.021320 19997371

[B16] Chen JJ Zhi L Yue S Gong QH Hybrid long-range surface plasmon-polariton modes with tight field confinement guided by asymmetrical waveguide Opt Express 2009 9 23603 23609 10.1364/OE.17.023603 20052069

[B17] Cai GX Luo M Xu HY Liu QH A slot-based surface plasmon-polariton waveguide with long-range propagation and superconfinement IEEE Photon J 2012 9 844 855

[B18] Chen L Zhang T Li X Huang WP Novel hybrid plasmonic waveguide consisting of two identical dielectric nanowires symmetrically placed on each side of a thin metal film Opt Express 2012 9 20535 20544 10.1364/OE.20.020535 23037100

[B19] Bian YS Gong QH Low-loss light transport at the subwavelength scale in silicon nano-slot based symmetric hybrid plasmonic waveguiding schemes Opt Express 2013 9 23907 23920 10.1364/OE.21.023907 24104301

[B20] Chen L Li X Wang GP A hybrid long-range plasmonic waveguide with sub-wavelength confinement Opt Commun 2013 9 400 404

[B21] Sun R Dong P Feng N Hong C Michel J Lipson M Kimerling L Horizontal single and multiple slot waveguides: optical transmission at λ = 1,550 nm Opt Express 2007 9 17967 17972 10.1364/OE.15.017967 19551093

[B22] Johnson PB Christie RW Optical constants of the noble metals Phys Rev B 1972 9 4370 4379 10.1103/PhysRevB.6.4370

[B23] Marinica DC Kazansky AK Nordlander P Aizpurua J Borisov AG Quantum plasmonic: nonlinear effects in the field enhancement of a plasmonic nanoparticle dimer Nano Lett 2012 9 1333 1339 10.1021/nl300269c 22320125

[B24] De Abajo FJ G Nonlocal effects in the plasmons of strongly interacting nanoparticles, dimers, and waveguides J Phys Chem C 2008 9 17983 17987 10.1021/jp807345h

[B25] Oulton RF Bartal G Pile DFP Zhang X Confinement and propagation characteristics of subwavelength plasmonic modes New J Phys 2008 9 1367 2630

